# Sensitivity Analysis of Weather Variables on Offsite Consequence Analysis Tools in South Korea and the United States

**DOI:** 10.3390/ijerph15051027

**Published:** 2018-05-18

**Authors:** Min-Uk Kim, Kyong Whan Moon, Jong-Ryeul Sohn, Sang-Hoon Byeon

**Affiliations:** Department of Health Science, Korea University, Anam-ro 145, Seongbuk-gu, Seoul 02841, Korea; qra00@korea.ac.kr (M.-U.K.); kwmoon@korea.ac.kr (K.W.M.); sohn1956@korea.ac.kr (J.-R.S.)

**Keywords:** offsite consequence analysis, KORA, ALOHA, weather variable, sensitivity analysis

## Abstract

We studied sensitive weather variables for consequence analysis, in the case of chemical leaks on the user side of offsite consequence analysis (OCA) tools. We used OCA tools Korea Offsite Risk Assessment (KORA) and Areal Location of Hazardous Atmospheres (ALOHA) in South Korea and the United States, respectively. The chemicals used for this analysis were 28% ammonia (NH_3_), 35% hydrogen chloride (HCl), 50% hydrofluoric acid (HF), and 69% nitric acid (HNO_3_). The accident scenarios were based on leakage accidents in storage tanks. The weather variables were air temperature, wind speed, humidity, and atmospheric stability. Sensitivity analysis was performed using the Statistical Package for the Social Sciences (SPSS) program for dummy regression analysis. Sensitivity analysis showed that impact distance was not sensitive to humidity. Impact distance was most sensitive to atmospheric stability, and was also more sensitive to air temperature than wind speed, according to both the KORA and ALOHA tools. Moreover, the weather variables were more sensitive in rural conditions than in urban conditions, with the ALOHA tool being more influenced by weather variables than the KORA tool. Therefore, if using the ALOHA tool instead of the KORA tool in rural conditions, users should be careful not to cause any differences in impact distance due to input errors of weather variables, with the most sensitive one being atmospheric stability.

## 1. Introduction

Chemical accidents are caused by worker errors, facility defects, or aging. Chemical leakage accidents can cause damage to humans or the environment [1]. Each country operates a chemical management system to prevent accidents caused by chemical substances. In 1982, the Seveso directive [2] was enacted in the European Union (EU) after the Seveso dioxin leak accident of 1976 in Italy. After the Texas explosion of 1989 in the United States (US), the US revised the Clean Air Act [3] in 1990. After the hydrogen fluoride (HF) leak of 2012 in South Korea (KOR), KOR enacted the Chemicals Control Act [4] in 2013 [5].

The US Clean Air Act requires that risk management plan (RMP) reports be submitted. The KOR Chemicals Control Act requires that offsite consequence analysis (OCA) reports be submitted. RMP and OCA reports include analysis of the impact on residents and the environment near the workplace, which consists of worst-case scenarios and alternative scenarios that can be expected from leakage accidents [6,7].

KOR’s National Institute of Chemical Safety (NICS) developed the Korea Offsite Risk Assessment (KORA) tool for OCA. In KOR, OCA tools being used include not only the KORA tool, but also the Areal Location of Hazardous Atmospheres (ALOHA) tool, developed by the United States Environmental Protection Agency (USEPA). The KORA and ALOHA tools were developed by applying various dispersion models, namely Gaussian models, SLAB (an atmospheric dispersion model for denser than air releases) models, and dense gas dispersion (DEGADIS) models [8,9]. Dispersion models require not only leakage data, but also weather data. Atmospheric dispersion is influenced by variables such as wind speed, atmospheric stability, and surface roughness. Dispersion models compute phenomena that disperse with physical space and time, such as weather conditions [10]. Analysis of these algorithms is generally not easy for those who use OCA tools. Furthermore, analyzing the source of OCA tools is not possible for the average user, unless they are a programmer.

RMP and OCA reports require accident scenarios. An accident scenario assumes an accident that could lead to an offsite impact through chemical leakage or serious injury to a worker. When a scenario is selected, weather data are used for determining the area’s weather information. Surface roughness takes the degree of obstacles such as buildings into account [11]. Input data of weather and surface roughness are determined by the user handling the OCA tool. Input data may vary depending on general weather patterns in the area, as well as the significance level of obstacles. Variation in input data of weather variables affects the impact distance [7,11].

Research related to OCA tools includes qualitative analysis studies of the models’ reliability improvement, as well as dispersion evaluation [12]. The aims have been to study the sensitivity of the SLAB model [13], to study land cover data to improve real time forecasting [14], and to study atmospheric dispersion evaluation characteristics [15]. The research on the KORA and ALOHA tools is devoted to predicting impact distances and deriving management plans based on actual accident cases [16,17,18,19,20]. Most people who use offsite analysis tools are general managers who manage chemical processes. Impact distance varies depending on input information of weather variables during those same processes. The general user of the KORA and ALOHA tools cannot easily analyze the sources and algorithms used in the tools. If we examine statistical results based on the user’s input information, we can see which factors play a significant role in deriving damage distances from the KORA and ALOHA tools. Therefore, in order to reduce the error of distance results, it is necessary to examine the correlation between weather variables on the user side. Therefore, in this study, we performed sensitivity analysis of OCA results according to weather variables, using user-side OCA tools.

## 2. Materials and Methods

### 2.1. Offsite Consequence Analysis

The OCA tools used were KORA version 2.0.0.0 [21] from KOR’s NICS, and ALOHA version 5.4.4 [22] from the USEPA. In terms of hazardous chemical substances, the frequency of accidents and the amounts used were ranked in the following order, based on accidents from 2009 to 2015: hydrogen chloride, nitric acid, ammonia, and hydrofluoric acid [23,24]. The concentrations of aqueous solutions were taken into account for the accident cases [25], along with concentration ranges supported by the KORA and ALOHA tools [21,22]. Therefore, the chemicals used were 28% ammonia (NH_3_), 35% hydrogen chloride (HCl), 50% hydrofluoric acid (HF), and 69% nitric acid (HNO_3_).

The scenarios were based on contents of leakage accident cases provided by the National Institute of Environmental Research [26]. Accident scenarios are defined as those in which 10-mm-diameter holes occur in storage tanks (operating temperature of 25 °C, operating pressure of 1.0 kg/cm^2^) containing 30 tons of hazardous chemicals in an aqueous solution, with leakage occurring for 10 min. The endpoint concentration for estimating the extent of the impact was applied to emergency response planning guidelines (ERPG)-2 values of the American Industrial Hygiene Association (AIHA) [27]. Table 1 shows input information of the KORA and ALOHA tools for modeling, according to scenarios tested.

The common weather variables in the KORA and ALOHA tools are wind speed, air temperature, humidity, and atmospheric stability. Variable conditions for wind speed, air temperature, and humidity were based on Korean weather information provided by the Korean Statistical Information Service (KOSIS) [28]. Air temperature was altered between −5 °C and 35 °C, at a rate of 5 °C. Wind speed was varied from 1 m/s to 16 m/s (maximum daily wind speed), in 1 m/s increments. Humidity was varied from 10% to 90%, at a rate of 10%. Atmospheric stability was altered from unstable (A) to stable (F). Surface roughness was divided into urban and rural [11]. We obtained a total of 640 results using the OCA tools. Table 2 shows a sample of some OCA results.

### 2.2. Sensitivity Analysis

Sensitivity analysis data utilized impact distances, which were the results of OCA by the KORA and ALOHA tools. Data reduction of impact distances was carried out using a Microsoft Excel spreadsheet [29]. The sample size used for sensitivity analysis was 640. There were no missing values. Sensitivity analysis was applied to regression analysis [30], which is a technique for determining the relationship between one or more independent variables and one dependent variable. Since impact distance is category-type data, the data for analysis were transformed into dummy variables (1 for coincidence and 0 for non-coincidence) [31], which were indicator variables representing each category for regression analysis. Table 3 shows a sample of some of the data sources converted to dummy variables. Regression analysis for the sensitivity analysis was performed using the Statistical Package for the Social Sciences (SPSS) program, version 24 [32], from the International Business Machines Corporation (IBM).

In this study, the independent variables were the chemicals used, air temperature, wind speed, humidity, atmospheric stability, surface roughness, and the OCA tools used, while the dependent variable was the impact distance. Therefore, the regression equation [33] derived for multiple regression analysis was as follows:

(1) $$\begin{array}{ll} \mathrm{Impact}\;\mathrm{distance}\; & =\;\mathrm{Constant}\;+\;\left(\beta_{1}\times \mathrm{Air}\;\mathrm{temperature}\right)+\left(\beta_{2}\times \mathrm{Wind}\;\mathrm{speed}\right)\\ & +\left(\beta_{3}\times \mathrm{Humidity}\right)+\left(\beta_{4}\;\times \;\mathrm{OCA}\;\mathrm{tool}\;\mathrm{used}\right)\\ & +\left(\beta_{5}\times \mathrm{Surface}\;\mathrm{roughness}\right)+\left(\beta_{6}\times \mathrm{Chemical}\;\mathrm{used}\right). \end{array}$$

## 3. Results

**Figure 1:**
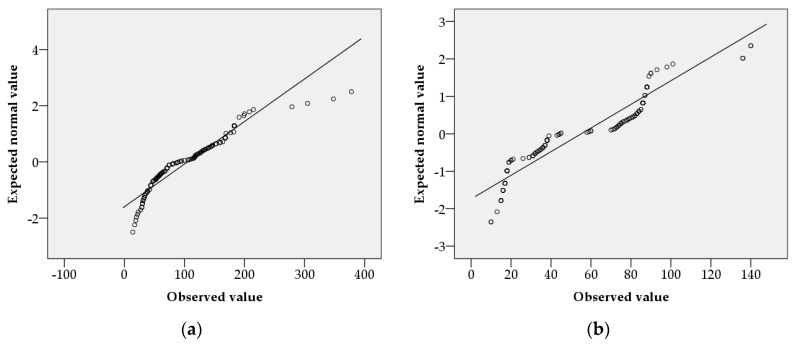
Normal quantile–quantile (Q–Q) plots of impact distances according to weather variables in urban conditions: (**a**) using the Areal Location of Hazardous Atmospheres (ALOHA) tool; (**b**) using the Korea Offsite Risk Assessment (KORA) tool.

**Figure 2:**
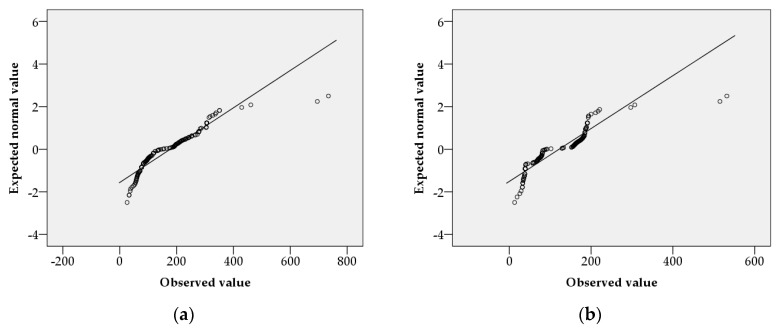
Normal Q–Q plots of impact distances according to weather variables in rural conditions: (**a**) using the ALOHA tool; (**b**) using the KORA tool.

**Figure 3:**
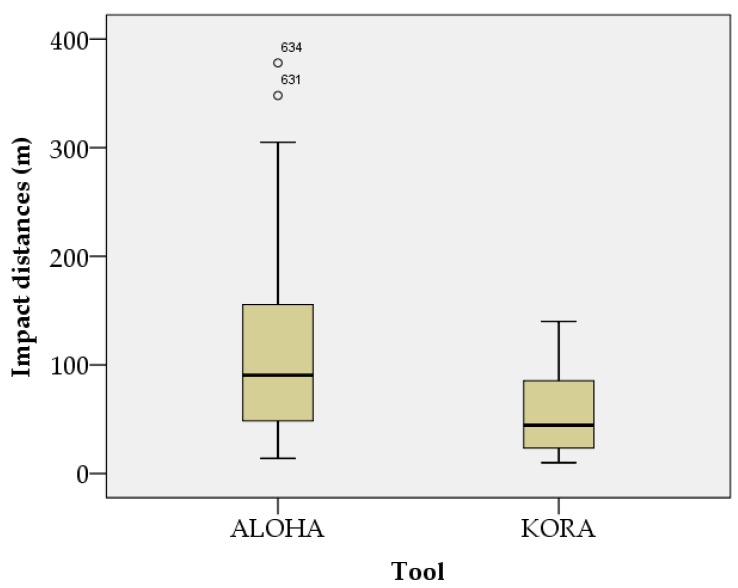
Box plot of impact distances according to weather variables in urban conditions.

**Figure 4:**
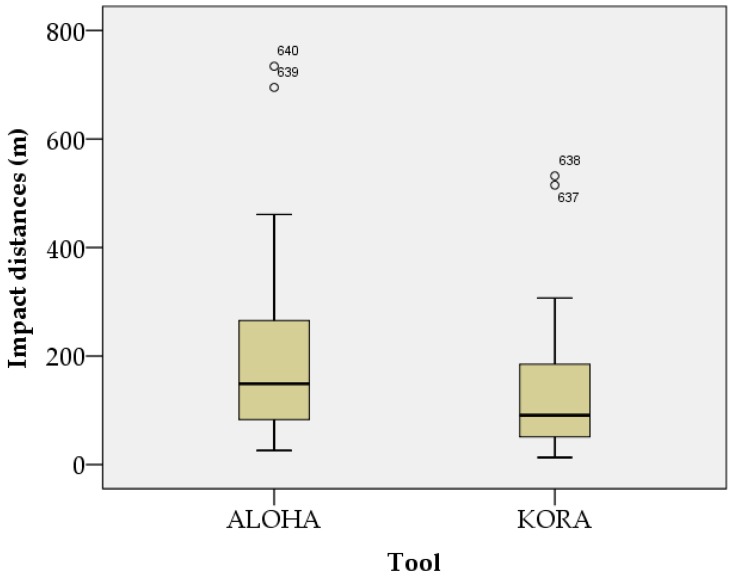
Box plot of impact distances according to weather variables in rural conditions.

The results of the normality test of the impact distances, according to weather variables in urban and rural conditions, are shown in Figure 1 and Figure 2. The impact distance data, according to weather variables in urban and rural conditions, followed a normal distribution. The results of the detection of outliers in the impact distances, according to weather variables in urban and rural conditions, are shown in Figure 3 and Figure 4. Outliers existed in all cases, except in impact distance data analyzed by the KORA tool in urban conditions.

Regression analysis for the sensitivity analysis between the impact distance and weather variables yielded results as follows. Table 4 shows the results of the regression analysis. The regression model showed significant correlation between variables (*p* < 0.001). Air temperature, wind speed, the OCA tool used, surface roughness, and the chemical used significantly affected the impact distance (*p* < 0.05), while the effect of humidity was not deemed significant (*p* = 0.112).

The regression equation for the sensitivity analysis according to weather variables, using the KORA and ALOHA tools in urban conditions, was as follows:

(2) $$\begin{array}{ll} \mathrm{Impact}\;\mathrm{distance} & =122.093+\left(-24.628\right)\times \mathrm{Air}\;\mathrm{temperature}+\left(-27.788\right)\\ & \times \mathrm{Wind}\;\mathrm{speed}+\left(-10.885\right)\times \mathrm{Humidity}+\left(-53.122\right)\times \mathrm{KORA}\\ & +\left(-67.766\right)\times \mathrm{Urban}+\left(32.556\right)\times 50\%\;\mathrm{HF}+\left(134.613\right)\\ & \times 28{\%\;\mathrm{NH}}_{3}+\left(126.775\right)\times 35\%\;\mathrm{HCl}. \end{array}$$

The sensitivity analysis results are shown in Table 5. Using the KORA tool, the impact distances for 28% NH_3_ were 255.91 m with atmospheric stability, 231.28 m with air temperature, and 228.12 m with wind speed. The impact distances for 35% HCl were 248.07 m with atmospheric stability, 223.45 m with air temperature, and 220.29 m with wind speed. The impact distances for 50% HF were 153.86 m with atmospheric stability, 129.23 m with air temperature, and 126.07 m with wind speed. The impact distances for 69% HNO_3_ were 121.30 m with atmospheric stability, 96.67 m with air temperature, and 93.51 m with wind speed. The impact distances for 28% NH_3_, according to weather variables, were the largest. When using the KORA tool, 28% NH_3_ was the most sensitive chemical in urban conditions. The impact distances according to atmospheric stability were the largest. Atmospheric stability was the most sensitive weather variable when using the KORA tool in urban conditions. Using the ALOHA tool, the impact distances for 28% NH_3_ were 309.03 m with atmospheric stability, 284.40 m with air temperature, and 281.25 m with wind speed. The impact distances for 35% HCl were 301.20 m with atmospheric stability, 276.57 m with air temperature, and 273.41 m with wind speed. The impact distances for 50% HF were 206.98 m with atmospheric stability, 182.35 m with air temperature, and 179.19 m with wind speed. The impact distances for 69% HNO_3_ were 174.42 m with atmospheric stability, 149.79 m with air temperature, and 146.63 m with wind speed. Sensitivity analysis results for the ALOHA tool were the same as those for the KORA tool. When using the ALOHA tool, 28% NH_3_, which had the greatest impact distances, was the most sensitive substance in urban conditions. Atmospheric stability, which had the greatest impact distances, was the most influential variable when using the ALOHA tool in urban conditions. Additionally, sensitivity analysis showed that impact distances using the ALOHA tool were larger than those using the KORA tool. Therefore, the ALOHA tool was more sensitive to weather variables than the KORA tool.

The regression equation for the sensitivity analysis according to weather variables, using the KORA and ALOHA tools in rural conditions, was as follows:

(3) $$\begin{array}{ll} \mathrm{Impact}\;\mathrm{distance} & =122.093+\left(-24.628\right)\times \mathrm{Air}\;\mathrm{temperature}+\left(-27.788\right)\\ & \times \mathrm{Wind}\;\mathrm{speed}+\left(-10.885\right)\times \mathrm{Humidity}+\left(-53.122\right)\times \mathrm{KORA}\\ & +\left(32.556\right)\times 50\%\;\mathrm{HF}+\left(134.613\right)\times 28{\%\;\mathrm{NH}}_{3}+\left(126.775\right)\\ & \times 35\%\;\mathrm{HCl}. \end{array}$$

The sensitivity analysis results are shown in Table 6. Using the KORA tool, the impact distances for 28% NH_3_ were 325.68 m with atmospheric stability, 301.05 m with air temperature, and 297.89 m with wind speed. The impact distances for 35% HCl were 317.84 m with atmospheric stability, 293.21 m with air temperature, and 290.05 m with wind speed. The impact distances for 50% HF were 223.62 m with atmospheric stability, 198.99 m with air temperature, and 195.83 m with wind speed. The impact distances for 69% HNO_3_ were 191.06 m with atmospheric stability, 166.44 m with air temperature, and 163.28 m with wind speed. When using the KORA tool, 28% NH_3_ was the chemical most affected by the weather variables. Among the meteorological variables, atmospheric stability had the greatest damage distances, making it the most influential variable when using the KORA tool in rural conditions. Sensitivity analysis using the KORA tool in rural conditions showed that the impact distances were larger in rural conditions than in urban conditions. Therefore, the KORA tool was more sensitive to weather variables in rural conditions than in urban conditions. Using the ALOHA tool, the impact distances for 28% NH_3_ were 378.80 m with atmospheric stability, 354.17 m with air temperature, and 351.01 m with wind speed. The impact distances for 35% HCl were 370.96 m with atmospheric stability, 346.33 m with air temperature, and 343.17 m with wind speed. The impact distances for 50% HF were 276.74 m with atmospheric stability, 252.11 m with air temperature, and 248.95 m with wind speed. The impact distances for 69% HNO_3_ were 244.19 m with atmospheric stability, 219.56 m with air temperature, and 216.40 m with wind speed. The sensitivity analysis results for the ALOHA tool in rural conditions were the same as for the KORA tool. When using the ALOHA tool, 28% NH_3_, which had the greatest impact distances, was the most sensitive substance in rural conditions. Atmospheric stability, which had the greatest impact distances among weather variables, was the most influential variable when using the ALOHA tool. Sensitivity analysis using the ALOHA tool in rural conditions showed that the impact distances were larger in rural conditions than in urban conditions. Therefore, the ALOHA tool was more sensitive to weather variables in rural conditions than in urban conditions. Additionally, the impact distances when using the ALOHA tool were larger than those when using the KORA tool. Therefore, the ALOHA tool was more sensitive to weather variables than the KORA tool.

## 4. Discussion

This study was relevant in terms of statistical analysis of the OCA tools used in KOR and the US, in accordance with weather variables on the user side. The parameters of the scenarios applied in this study may be limited to each facility. This is because the scenarios selected situations with the greatest impact distances following chemical leakages due to equipment damage, breakage, fracture. [34,35]. Moreover, temperature, wind speed, humidity, and atmospheric stability interact with each other in actual atmospheric dispersion [36,37]. However, in this study, these interactions were not considered when analyzing the independent sensitivity of OCA tools to common weather variables.

The sample size used for multiple regression analysis was 640. The normality test used a quantile–quantile (Q–Q) plot. If the number of samples is large, the normality of its distribution can be assumed by the representative approximation theory and the central limit theorem [38]. Central limit theorems have been used in various fields of research [39,40,41,42]. One study in the field of info-communications assumed normality by using the central limit theorem to estimate sensor location in a non-uniform network environment [42], as the central limit theorem proved that many sensor network environments meet a normal distribution model. Therefore, in this study, the central limit theorem could be used to prove that a sufficiently large sample (*n* ≥ 30) conformed to a normal distribution. Box plots were used to find outliers, of which there were six. Outliers occurred when measurement values were correctly observed, but they were rare cases. In other words, outliers are impact distances that rarely occur in OCA. Outliers are not objects that need to be removed, but rather objects that contain important information about the entire dataset [43]. Not all outliers are a problem [43]. The analysis of outliers in categorical data should be subject to separate subjective considerations [43]. In this study, we did not eliminate outliers, as they were realistic outcomes when using the KORA and ALOHA tools. Therefore, the regression equation used in the sensitivity analysis was a reflection of reality.

Sensitivity analysis of the KORA and ALOHA tools for each substance, according to weather conditions, indicated that 28% NH_3_ showed the greatest damage distances. Moreover, sensitivity was found to decrease in the following order: 35% HCl, 50% HF, and 69% HNO_3_. Assuming that emissions per hour and evaporation remain constant, along with temperature, wind speed, and humidity, specific gravity is a physicochemical factor that affects the diffusion of chemicals [37,44,45]. The specific gravity of 28% NH_3_ is lower than one, and is lower than that of 35% HCl, 50% HF, and 69% HNO_3_. The specific gravity of 35% HCl is lower than that of 50% HF and 69% HNO_3_. The specific gravity of 50% HF is lower than that of 69% HNO_3_ [46]. Therefore, we can objectively observe, using statistical analysis, that substances with low specific gravity are more sensitive to weather variables than those with higher specific gravity.

The effect of humidity on impact distances was not significant (*p* = 0.112). In other words, there was no change in impact distances according to humidity. Unlike humidity, temperature, wind speed, and atmospheric stability affected damage distances significantly (*p* < 0.05). The impact distances in urban and rural conditions were largest according to atmospheric stability, while those according to air temperature were greater than those according to wind speed. Moreover, the ALOHA tool had larger impact distances than the KORA tool. Therefore, atmospheric stability in urban and rural areas was the most sensitive factor for the KORA and ALOHA tools, while air temperature was a more sensitive factor than wind speed. The KORA and ALOHA tools were more sensitive to atmospheric stability, air temperature, and wind speed in rural conditions than in urban conditions. In addition, the ALOHA tool was more affected by atmospheric stability, air temperature, and wind speed than the KORA tool. Users of the KORA and ALOHA tools need to be careful to not commit input errors in atmospheric stability, which was the most sensitive factor, so as to reduce variation in damage distances due to those errors. In particular, the ALOHA tool was more sensitive to atmospheric stability, air temperature, and wind speed than the KORA tool. Therefore, users should be more careful to not commit input errors regarding weather variables when using the KORA tool than when using the ALOHA tool.

## 5. Conclusions

Regression analysis showed that the KORA and ALOHA tools had large impact distances when using the following chemicals, in descending order: 28% NH_3_, 35% HCl, 50% HF, and 69% HNO_3_. Therefore, 28% NH_3_ was the most sensitive chemical to weather variables. Meanwhile, 35% HCl was a more sensitive chemical than 50% HF and 69% HNO_3_, while 50% HF was a more sensitive chemical than 69% HNO_3_. From the results of the sensitivity analysis of weather variables, humidity was found to neither affect the KORA nor ALOHA tools. Unlike humidity, atmospheric stability was the most sensitive factor for the KORA and ALOHA tools, with air temperature being a more sensitive factor than wind speed. The weather variables (atmospheric stability, air temperature, and wind speed) were more sensitive factors in rural conditions than in urban conditions, while the ALOHA tool was more sensitive than the KORA tool. Therefore, users of the ALOHA tool need to be careful to not affect impact distances due to input errors in atmospheric stability in rural conditions, as this was the most sensitive weather variable in OCA.

## Figures and Tables

**Table 1:** Input data provided to the tools.

Tools	Classification	Information
**Areal Location of Hazardous Atmospheres (ALOHA)**	Measurement height above ground	3 m
	Cloud cover	Partly cloudy
	Source	Puddle
	Puddle diameter	10 m
	Mass of puddle	0.54 ton (50% hydrofluoric acid, HF), 0.42 ton (28% ammonia, NH_3_) 0.54 ton (35% hydrogen chloride, HCl), 0.6 ton (69% nitric acid, HNO_3_)
	Ground type	Concrete
**Korea Offsite Risk Assessment (KORA)**	Equipment appearance	Vertical cylinder (drum)
	Equipment diameter	3 m
	Equipment height	6 m
	Storage amount	30 ton
	Operating pressure	1.0 kg/cm^2^
	Bonded pipe diameter	50 mm
	Leakage type	Storage tank leakage
	Height of leakage hole	0.5 m
	Diameter of leakage hole	10 mm

**Table 2:** Offsite consequence analysis (OCA) results for 50% HF and 28% NH_3_ by weather variables (*N* = 640).

Weather Variables		50% HF				28% NH_3_			
		KORA		ALOHA		KORA		ALOHA	
		Urban (m)	Rural (m)	Urban (m)	Rural (m)	Urban (m)	Rural (m)	Urban (m)	Rural (m)
**Air Temperature**	−5 °C	36	77	38	71	84	181	95	156
	0 °C	36	77	42	77	84	182	106	175
	5 °C	36	78	47	84	85	184	118	194
	10 °C	37	79	53	91	86	186	132	217
	15 °C	37	80	58	99	87	188	147	244
	20 °C	37	80	64	108	88	189	164	273
	25 °C	38	81	71	119	88	191	183	306
	30 °C	38	82	81	136	89	193	200	336
	35 °C	38	82	92	153	90	194	208	351
**Wind Speed**	1 m/s	43	92	88	145	101	217	215	339
	2 m/s	39	85	80	133	93	200	199	318
	3 m/s	38	81	71	119	88	191	183	306
	4 m/s	36	78	68	114	86	185	169	283
	5 m/s	35	76	66	111	83	180	160	266
	6 m/s	35	75	64	108	82	176	152	252
	7 m/s	34	73	63	106	80	173	145	241
	8 m/s	34	72	61	104	79	170	139	231
	9 m/s	33	71	60	102	78	168	135	222
	10 m/s	33	70	59	100	77	166	130	215
	11 m/s	32	70	58	99	76	164	126	208
	12 m/s	32	69	57	98	75	162	122	203
	13 m/s	32	68	56	97	75	161	119	197
	14 m/s	31	68	55	96	74	160	116	193
	15 m/s	31	67	54	95	73	158	114	189
	16 m/s	31	67	53	94	73	157	111	184
**Humidity**	10%	38	81	71	119	88	191	183	306
	20%	38	81	71	119	88	191	183	306
	30%	38	81	71	119	88	191	183	306
	40%	38	81	71	119	88	191	183	306
	50%	38	81	71	119	88	191	183	306
	60%	38	81	71	119	88	191	183	306
	70%	38	81	71	119	88	191	183	306
	80%	38	81	71	119	88	191	183	306
	90%	38	81	71	119	88	191	183	306
**Atmospheric Stability**	A	20	26	33	37	45	59	81	91
	B	20	39	38	56	45	90	95	138
	C	26	58	49	83	59	133	128	207
	D	38	81	71	119	88	191	183	306
	E	60	130	118	198	140	307	305	461
	F	60	221	145	351	140	532	378	734

**Table 3:** Some of the data sources from the OCA results converted to dummy variables (*N* = 604).

50% HF	28% NH_3_	30% HCl	69% HNO_3_	KORA	ALOHA	Urban	Rural	AT	WS	HU	AS	ID (m)
1	0	0	0	1	0	1	0	1	0	0	0	36
1	0	0	0	1	0	1	0	1	0	0	0	36
1	0	0	0	1	0	1	0	1	0	0	0	36
1	0	0	0	1	0	1	0	1	0	0	0	37
1	0	0	0	1	0	1	0	1	0	0	0	37
1	0	0	0	1	0	1	0	1	0	0	0	37
1	0	0	0	1	0	1	0	1	0	0	0	38
1	0	0	0	1	0	1	0	1	0	0	0	38
1	0	0	0	1	0	1	0	1	0	0	0	38
1	0	0	0	1	0	1	0	0	1	0	0	43
1	0	0	0	1	0	1	0	0	1	0	0	39
1	0	0	0	1	0	1	0	0	1	0	0	38
1	0	0	0	1	0	1	0	0	1	0	0	36
1	0	0	0	1	0	1	0	0	1	0	0	35
1	0	0	0	1	0	1	0	0	1	0	0	35
1	0	0	0	1	0	1	0	0	1	0	0	34
1	0	0	0	1	0	1	0	0	1	0	0	34
1	0	0	0	1	0	1	0	0	1	0	0	33
1	0	0	0	1	0	1	0	0	1	0	0	33
1	0	0	0	1	0	1	0	0	1	0	0	32
1	0	0	0	1	0	1	0	0	1	0	0	32
1	0	0	0	1	0	1	0	0	1	0	0	32
1	0	0	0	1	0	1	0	0	1	0	0	31
1	0	0	0	1	0	1	0	0	1	0	0	31
1	0	0	0	1	0	1	0	0	1	0	0	31
1	0	0	0	1	0	1	0	0	0	1	0	38
1	0	0	0	1	0	1	0	0	0	1	0	38
1	0	0	0	1	0	1	0	0	0	1	0	38
1	0	0	0	1	0	1	0	0	0	1	0	38
1	0	0	0	1	0	1	0	0	0	1	0	38
1	0	0	0	1	0	1	0	0	0	1	0	38
1	0	0	0	1	0	1	0	0	0	1	0	38
1	0	0	0	1	0	1	0	0	0	1	0	38
1	0	0	0	1	0	1	0	0	0	1	0	38
1	0	0	0	1	0	1	0	0	0	0	1	20
1	0	0	0	1	0	1	0	0	0	0	1	20
1	0	0	0	1	0	1	0	0	0	0	1	26
1	0	0	0	1	0	1	0	0	0	0	1	38
1	0	0	0	1	0	1	0	0	0	0	1	60
1	0	0	0	1	0	1	0	0	0	0	1	60
1	0	0	0	1	0	0	1	1	0	0	0	77
1	0	0	0	1	0	0	1	1	0	0	0	77

AT: air temperature. WS: wind speed. HU: humidity. AS: atmospheric stability. ID: impact distance.

**Table 4:** Regression analysis for the sensitivity analysis of weather variables.

Variable	β	t
Air temperature	−24.628	−3.602 *
Wind speed	−27.788	−4.475 *
Humidity	−10.885	−1.592
KORA	−53.122	−12.950 *
Urban	−69.766	−17.008 *
50% HF	32.556	5.612 *
28% NH_3_	134.613	23.205 *
35% HCl	126.775	21.854 *
Constant	122.093	17.428 *
F Value	161.716 *	
Adjusted R^2^	0.668	

* *p* < 0.05.

**Table 5:** Sensitivity analysis of weather variables according to chemicals in urban conditions.

Chemicals	Weather Variables	KORA (m)	ALOHA (m)
28% NH_3_	Atmospheric stability	255.91	309.03
	Air temperature	231.28	284.40
	Wind speed	228.12	281.25
35% HCl	Atmospheric stability	248.07	301.20
	Air temperature	223.45	276.57
	Wind speed	220.29	273.41
50% HF	Atmospheric stability	153.86	206.98
	Air temperature	129.23	182.35
	Wind speed	126.07	179.19
69% HNO_3_	Atmospheric stability	121.30	174.42
	Air temperature	96.67	149.79
	Wind speed	93.51	146.63

**Table 6:** Sensitivity analysis of weather variables according to chemicals in rural conditions.

Chemicals	Weather Variables	KORA (m)	ALOHA (m)
28% NH_3_	Atmospheric stability	325.68	278.80
	Air temperature	301.05	354.17
	Wind speed	297.89	351.01
35% HCl	Atmospheric stability	317.84	370.96
	Air temperature	293.21	346.33
	Wind speed	290.05	343.17
50% HF	Atmospheric stability	223.62	276.74
	Air temperature	198.99	252.11
	Wind speed	195.83	248.95
69% HNO_3_	Atmospheric stability	191.06	244.19
	Air temperature	166.44	219.56
	Wind speed	163.28	216.40
